# Interactions of Biocidal Polyhexamethylene Guanidine Hydrochloride and Its Analogs with POPC Model Membranes

**DOI:** 10.3390/polym9100517

**Published:** 2017-10-17

**Authors:** Xuliang Luo, Ziran Jiang, Niya Zhang, Zixin Yang, Zhongxin Zhou

**Affiliations:** 1 Key Lab of Agricultural Animal Genetics, Breeding and Reproduction of Ministry of Education, College of Animal Sciences & Technology, Huazhong Agriculture University, 1 Shizishan Street, Wuhan 430070, China; hzauluoxuliang@163.com (X.L.); jiangziran163@163.com (Z.J.); zhangniya@mail.hzau.edu.cn (N.Z.); 2 The Cooperative Innovation Center for Sustainable Pig Production, Huazhong Agriculture University, 1 Shizishan Street, Wuhan 430070, China; 3 College of Sciences, Huazhong Agriculture University, 1 Shizishan Street, Wuhan 430070, China; zixinyang@mail.hzau.edu.cn

**Keywords:** polyhexamethylene guanidine hydrochloride, membrane, POPC, hemolytic activity, dye leakage

## Abstract

The bacterial membrane-targeted polyhexamethylene guanidine hydrochloride (PHGH) and its novel analog polyoctamethylene guanidine hydrochloride (POGH) had excellent antimicrobial activities against antibiotics-resistant bacteria. However, the biocompatibility aspects of PHGH and POGH on the phospholipid membrane of the eukaryotic cell have not yet been considered. Four chemically synthesized cationic oligoguanidine polymers containing alkyl group with different carbon chain lengths, including PHGH, POGH, and their two analogs, were used to determine their interactions with zwitterionic 1-palmitoyl-2-oleoyl-sn-glycero-3-phosphocholine (POPC) phospholipids vesicles mimicking the eukaryotic cell membrane. Characterization was conducted by using bactericidal dynamics, hemolysis testing, calcein dye leakage, and isothermal titration calorimetry. Results showed that the gradually lengthened alkyl carbon chain of four oligoguanidine polymers increased the biocidal activity of the polymer, accompanied with the increased hemolytic activity, calcein dye leakage rate and the increased absolute value of the exothermic effect of polymer-POPC membrane interaction. The thermodynamic curve of the polymer-POPC membrane interaction exhibited a very weak exothermic effect and a poorly unsaturated titration curve, which indicated that four guanidine polymers had weak affinity for zwitterionic POPC vesicles. Generally, PHGH of four guanidine polymers had high biocidal activity and relatively high biocompatibility. This study emphasized that appropriate amphiphilicity balanced by the alkyl chain length, and the positive charge is important factor for the biocompatibility of cationic antimicrobial guanidine polymer. Both PHGH and POGH exhibited destructive power to phospholipid membrane of eukaryotic cell, which should be considered in their industry applications.

## 1. Introduction

The mechanism of the antimicrobial cationic amphiphile selectively targeting the bacterial lipid membrane rather than the eukaryotic cell has been yet not clearly understood [1,2,3]. The bacterial membrane is considered as the very promising target of antimicrobials against multidrug-resistant Gram-negative pathogens [4,5,6,7]. In particular, the membrane-targeted antimicrobial cationic amphiphiles are relatively difficult for bacteria to develop resistance against [1,8].

Membrane targeted guanidine-based amphiphilic polymers show excellent antibacterial [9,10,11,12], antifungal [13], and antiviral [14,15] activities. Interestingly, cumulative developments show that antimicrobial molecule containing multiple guanidinium groups is endowed with antimicrobial activities [16,17] against one or more of six high-priority dangerous antibiotic-resistant ESKAPE pathogens [18] in clinical practices, including multidrug resistant *Pseudomonas aeruginosa* (MDR-PA), methicillin-resistant *Staphylococcus aureus* (MRSA), multidrug resistant *Acinetobacter* species, vancomycin resistant *Enterococcus faecium*, *Enterobacter* species, Extended-Spectrum *β*-Lactamases-producing *Klebsiella* species.

As we have previously reported, polyhexamethylene guanidine hydrochloride (PHGH) and its three novel analogs, including polybutamethylene guanidine hydrochloride, polyoctamethylene guanidine hydrochloride (POGH) and poly(m-xylylene guanidine hydrochloride), exhibited extensive in vitro antimicrobial activities against 370 clinical strains, especially 96 antibiotics-resistant isolates [19]. It was found that PHGH was especially efficient against MRSA and methicillin resistant-coagulase-negative staphylococci with a minimum inhibitory concentration (MIC) range of 1–8 mg/L [19,20,21]. POGH, the novel oligoguanidine, had a significantly lower MIC values range (0.5–16 mg/L) against 370 antibiotics-susceptible and -resistant clinical strains compared to PHGH (MIC, 1–64 mg/L) and chlorhexidine digluconate (MIC, 2–64 mg/L), another widely used disinfectant in hospitals [19]. Importantly, POGH displayed excellent activity against MRSA (MIC, 1–8 mg/L) and methicillin-resistant coagulase-negative staphylococci (MIC, 1–2 mg/L), vancomycin-resistant *Enterococcus faecium* (MIC, 2–4 mg/L), multidrug-resistant *Pseudomonas aeruginosa* (MIC, 8–16 mg/L), ceftazidime-resistant *Citrobacter* spp. (MIC, 1–4 mg/L) and *Enterobacter* spp. (MIC, 2–4 mg/L) [19].

The main antimicrobial mechanism of guanidine-based cationic polymer has been demonstrated to mainly disrupt the cytoplasmic phospholipid membrane of the bacteria cell [22,23]. However, for the safety and biocompatibility aspects, the effects of PHGH and POGH on the phospholipid membrane of the eukaryotic cell have not yet been considered. The hemolytic activity of the antimicrobial polymer on human erythrocytes is usually used to evaluate its biocompatibility because the membrane of red blood cells is extremely fragile [24]. The details of polymer-membrane interactions have been elucidated based on phospholipid model membrane system [25] and biophysical techniques, for example, isothermal titration calorimetry (ITC) [22], encapsulated fluorescence dye leakage test [22]. 1-palmitoyl-2-oleoyl-sn-glycero-3-phosphocholine (POPC) phospholipid vesicles have been used as a model of the eukaryotic membrane [26].

Therefore, to evaluate the biocompatibility, four synthesized biocidal guanidine hydrochloride polymers with different alkyl chain lengths, including PHGH, POGH and their two analogs, were used to investigate their interactions with 1-palmitoyl-2-oleoyl-sn-glycero-3-phosphocholine (POPC) phospholipids vesicles. Characterization was performed by using bactericidal dynamics, hemolysis testing, fluorescence dye leakage, and isothermal titration calorimetry.

## 2. Materials and Methods

### 2.1. Reagents

Phospholipids, 1-palmitoyl-2-oleoyl-sn-glycero-3-phosphocholine (POPC), were purchased from Avanti Polar Lipids Inc. (Alabaster, AL, USA). The whole blood of human was available from Shanghai (Red Cross) Blood Center (WHO Collaborating Center for Blood Transfusion Servies, Geneva, Switzerland). Tris(hydroxymethyl)aminoethane were purchased from Sigma-Aldrich Co. (St. Louis, MO, USA). These chemicals were used as received. All used salts were of analytical purity. Double distilled water was used in all experiments.

### 2.2. Oligoguanidine Polymers

**Figure 1:**
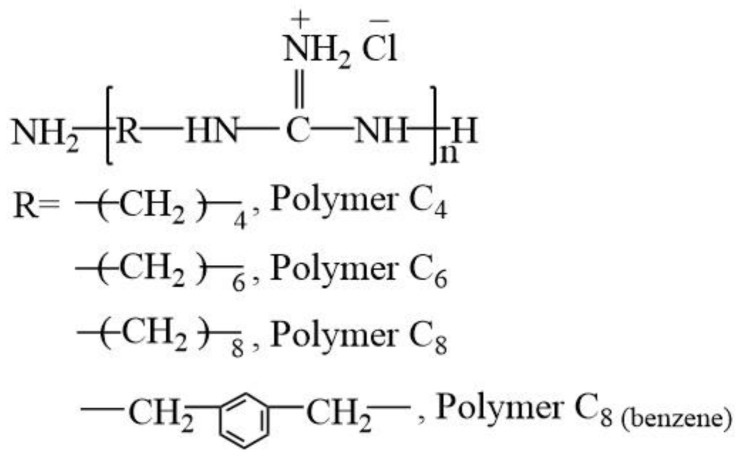
The chemical structure of four oligoguanidine polymer analogs: Polybutamethylene guanidine hydrochloride (Polymer C_4_); Polyhexamethylene guanidine hydrochloride (PHGH, Polymer C_6_); Polyoctanethylene guanidine hydrochloride (POGH, Polymer C_8_); Poly(m-xylylene guanidine hydrochloride) (Polymer C_8(benzene)_).

Four oligoguanidine polymer analogs, polyhexamethylene guanidine hydrochloride (PHGH, Polymer C_6_), polyoctanethylene guanidine hydrochloride (POGH, Polymer C_8_), and their two analogs, including polybutamethylene guanidine hydrochloride (Polymer C_4_) and poly(m-xylylene guanidine hydrochloride) (Polymer C_8(benzene)_), were provided by Dr. Dafu Wei in School of Materials Science and Engineering, East China University of Science and Technology (Shanghai, China). These oligoguanidine polymers were synthesized as reported in Materials Science and Engineering C 31 (2011) 1836–1843 [19], and their structures are shown in Figure 1. The number-average molecular weight (*M*_n_), weight-average molecular weight (*M*_w_), and polydispersity coefficient (*D*_p_) of Polymer C_4_ are 362, 405, 1.12, respectively. The values of *M*_n_, *M*_w_ and *D*_p_ of Polymer C_6_ are 481, 576, 1.2, respectively. The values of *M*_n_, *M*_w_ and *D*_p_ of Polymer C_8_ are 457, 512, 1.12, respectively. The values of *M*_n_, *M*_w_ and *D*_p_ of Polymer C_8(benzene)_ are 503, 634, 1.26, respectively.

### 2.3. Bacterial Strains

MDR-PA clinical strains were provided by Huashan Hospital, ShanghaiMedical College, Fudan University (Shanghai, China).The Vitek automated identification system (BioMérieux, Marcy l’Etoile, France) were used to identify MDR-PA strains and the API-GN system (BioMérieux, Marcy l’Etoile, France) confirmation followed.

### 2.4. Bactericidal Dynamics

Time-killing curves of four oligoguanidine polymer analogs were determined against MDR-PA. Four polymers were dissolved in 10 mM phosphate buffer solution (PBS, pH 7.4) with a final pH 7.4 to prepare the reserved solutions. 20 μL MDR-PA aliquot was added to a 250 mL conical flask containing 40 mL of Luria-Bertani (LB) broth. Bacteria were cultured on a shaker overnight (200 rpm at 37 °C). The next morning, a 200 μL bacterial medium was added to a 250 mL conical flask containing 40 mL of fresh LB medium. The MDR-PA bacteria were continuously cultured to an optical density of 1 at a wavelength of 600 nm (OD_600_). The cultures were further suspended, centrifuged and resuspended with 10 mM PBS (pH 7.4) for three cycles. The centrifugation rate was 10,000 revolutions per minute (rpm), and the centrifugation time was 1 min. Finally, the 6.25 × 10^6^ CFU/mL bacterial suspension was incubated, respectively, with four guanidine hydrochloride polymer analogs (Polymer C_4_, C_6_, C_8_, C_8(benzene)_) for 0.5, 1, 1.5, 2 and 2.5 h at 30 °C. After various time points, 20 μL aliquots were withdrawn, immediately followed by a 1:100 dilution in 10 mM PBS, which was used to arrest treatment, and then a serial tenfold dilution was applied. Diluted MDR-PA cell suspensions of 8 μL from each dilution was spread on Muller Hinton broth-agar plates, and the number of live bacteria after 24 h at 37 °C was counted. The MDR-PA cell suspensions with an optical density of 0.35 at 600 nm corresponded to 2.5 × 10^8^ colony forming unit (CFU) per milliliter based on the plate counts.

### 2.5. Hemolysis Testing

Fresh human erythrocytes were obtained by centrifuging whole blood at 3000 revolutions per minute (rpm) for 10 min (for the removing of the plasma and white blood cells). The red blood cells (RBCs) were diluted with Tris buffer saline (TBS, 10 mM Tris, 150 mM NaCl, pH 7.4) to yield RBC stock solution of 0.3% (*v/v*). Four polymers were dissolved in TBS with final pH 7.4, then were diluted with TBS to different concentrations (2–1000 μg/mL). Furthermore, above polymer solutions at different concentrations were pipetted into test tubes. The hemolysis was performed in a tube containing 2.5 mL RBCs + 2.5 mL (TBS + polymer). All test tubes were incubated in water bath at 37 °C for 1 h, then centrifuged at 3000 rpm for 10 min to settle the unruptured RBCs. Hemolysis was monitored by measuring the absorbance of hemoglobin released due to cell lysis in the supernatant at 414 nm (Unico^TM^, UV-2100, UNICO Instruments Co., Ltd., Shanghai, China). The polymers had a comparatively small absorbance (about optical density of 0.03) at 414 nm. Positive control (100% hemolysis) was obtained by adding RBCs and triton X-100 to achieve the complete lysis of the blood cells (5 mL = 2.5 mL RBCs + 0.25 mL 1% triton X-100 + 2.25 mL TBS). Negative control (0% hemolysis) was obtained by adding 2.5 mL RBCs to 2.5 mL TBS. The hemolysis rate (%) was calculated using the following equation:

Hemolysis Rate % = 100 × (OD_assay_ − OD_negative_)/(OD_positive_ − OD_negative_).

OD was defined as optical density value at the assay wavelength.

### 2.6. Polymer-Induced Leakage Assays of Fluorescence Dye

Large unilamellar vesicles (LUVs) for fluorescence dye leakage were prepared by the extrusion method. About 10 mg POPC dry lipids were dissolved in 10 mL chloroform/methanol mixture (*v/v*, 2:1) in a 25 mL eggplant-type flask, respectively. After the lipids were dissolved, the chloroform/methanol mixture were removed by rotary evaporator in a 25 °C water bath to form a uniform lipid film in the eggplant-type flask of 25 mL. Trace amounts of the chloroform/methanol mixture were eliminated by placing the eggplant-type flask under vacuum overnight. The dried lipid film in the eggplant-type flask was hydrated by using 1 mL Tris buffer containing calcein dye (40 mM calcein, 10 mM Tris, pH 7.4) at 25 °C (at least 10 °C above the *T*_m_ of the phospholipids), and the buffer were vortexed for about 30 min until the entire lipid film was eliminated from the wall of the flask and a homogenous multilamellar lipid vesicles (MLVs) suspension was formed. The MLVs suspension was subjected to five freeze/thaw cycles in the liquid nitrogen and the warm water. Then the suspension was subjected to 15 extrusion cycles through a mini-extruder equipped with a polycarbonate filter membrane with 1 μm and 400 nm pore diameter, respectively, to form the LUVs at room temperature. The unencapsulated calcein was removed by using the gel filtration method on a 1.6 cm × 40 cm Sephadex G-50 superfine (~10 g) column with a 0.5 mL/min flow rate, the collected fractions in the void volume were stored at 4 °C and diluted to measuring for up to 7 days. The concentration of phospholipid was measured by using the phosphate analysis method [27], and the LUVs were diluted to appropriate concentration before measurement.

Fluorescence leakage measurements were completed in the SLM Aminco Bowman Series II spectrofluorimeter (SLM Instruments, New York, NY, USA), an excitation wavelength of 490 nm and an emission wavelength of 515 nm with 5-nm bandwidth slits. A quartz cuvette (2 mL total volume) containing calcein-loaded LUVs of 20 μM final concentration was prepared. Leakage was initiated when the polymer with a final concentration of 10 μg/mL was added, and the fluorescence was recorded at 30 °C. For the measurement of 100% dye release, 20% Triton X-100 (50 μL) was added to dissolve the vesicles without polymer addition. The percentage of dye leakage induced by the oligoguanidine polymer was calculated as follows: dye leakage (%) = 100 × (*F* − *F*_0_)/(*F*_t_ − *F*_0_), where *F* is the fluorescence intensity achieved by oligoguanidine polymer addition, *F*_0_ is the fluorescence intensities without any oligoguanidine polymer addition or Trition addition, and *F*_t_ is the fluorescence intensities induced by the addition of 20% Triton X-100.

### 2.7. Isothermal Titration Calorimetry

LUVs for isothermal titration calorimetry were made as calcein-loaded LUVs with a minor modification. About 40 mg POPC dry lipids was used, and the formed lipid film was hydrated with 2.2 mL Tris buffer saline (TBS, 10 mM Tris, 150 mM NaCl, pH 7.4).

Titration experiments were completed in a VP-ITC instrument (MicroCal Inc., Piscataway, NJ, USA). All solutions were degassed under vacuum for 15 min before the measurement was performed. Each oligoguanidine polymer solution, at a concentration of 75 μg/mL dissolved in TBS (adjusted final pH 7.4), was placed in the 1.414 mL calorimeter cell. LUV suspension (15 mM) was placed in the 295.66 μL syringe and injected over 20 s in aliquots of 10 μL (the first injection was 3 μL) with 300 s intervals between the individual injections. For equilibrium, the syringe was assembled into the calorimeter cell of the VP-ITC instrument while stirring at 300 rpm. The temperature (set at 30 °C) of the calorimeter cell and the baseline heat signal (set at 15 μcal/s) were usually stabilized within 15–30 min. To account for the dilution heat, control experiment was performed by titrating lipid vesicles into the TBS solution in the absence of oligoguanidine polymer. Data acquisition and analysis were completed using Microcal Origin software.

### 2.8. Toxicological Studies

The acute toxic effects of polyoctamethylene monoguanidine hydrochloride (POGH) and the well-known polyhexamethylene monoguanidine hydrochloride (PHGH) after peroral administration to rats were simply determined based on the method of safety evaluation of AKACID Plus^®^ [28], which is a commercial member of the polymeric guanidine family of disinfectants. For every one of both polymers, initially the experiment was carried out with one group consisting of two female and two male mice given a dose of 200 milligram (mg) of polymer per kilogram (kg) of body weight. After observations for two days, added two groups consisting of two female and two male mice were performed, the polymer dose was increased to 500 and 800 mg/kg of body weight, respectively. All mice were killed by inhalation of CO_2_ on day 14 and subjected to a gross necropsy examination. The study was approved by the The Scientific Ethic Committee of Huazhong Agericulture University (HZAUMO-2016-046).

## 3. Results

### 3.1. Polymer C_4_, C_6_, C_8_ Had Systematically Increased Antimicrobial Activity with an Increase in the Alkyl Chain Length of the Polymer

**Figure 2:**
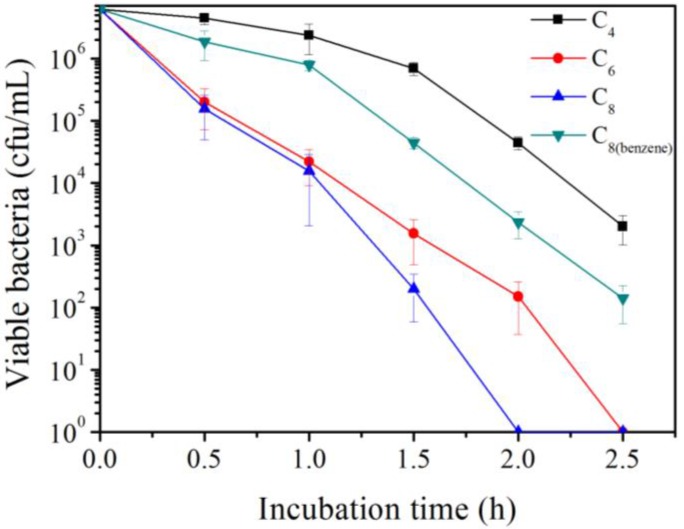
The time-kill curve of synthesized Polymer C_4_, C_6_, C_8_, C_8(benzene)_ against multidrug-resistant *Pseudomonas aeruginosa*. Approximately 6.25 × 10^6^ CFU/mL bacterial cells were incubated, respectively, with 32 μg/mL polymer at 25 °C for 0.5, 1, 1.5, 2 and 2.5 h.

Based on MIC tests, guanidine-based hydrochloride polymers have been reported to display antimicrobial activities against antibiotics-resistant bacteria [19,21]. Multidrug-resistant *Pseudomonas aeruginosa* (MDR-PA) is considered as one of six high-priority dangerous ESKAPE pathogens in clinical practice [29,30]. Furthermore, in this study, the time-kill curves (bactericidal dynamics) of synthesized four oligoguanidine hydrochloride polymers (Polymer C_4_, C_6_, C_8_, C_8(benzene)_) against MDR-PA were performed. Figure 2 showed that Polymer C_4_, C_6_, C_8_ had systematically increased bactericidal activity with an increase in the alkyl chain length of the polymer, and Polymer C_8_ had higher bactericidal activity than Polymer C_8(benzene)_.

### 3.2. Hemolytic Property

**Figure 3:**
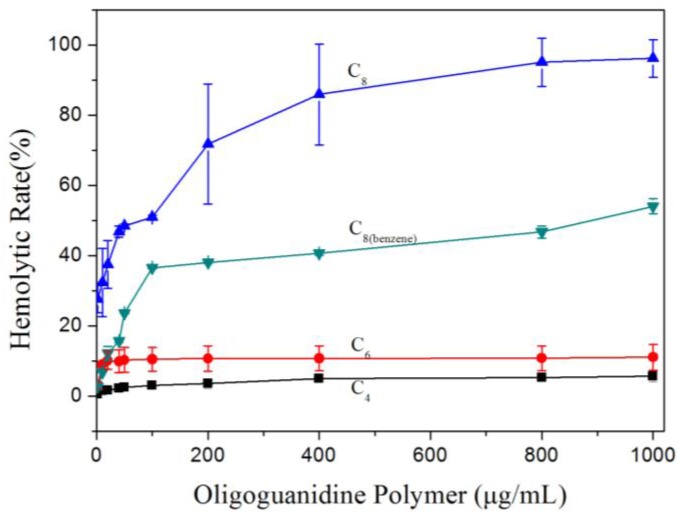
Hemolytic property of synthesized oligoguanidine Polymer C_4_, C_6_, C_8_, C_8(benzene)_ against human erythrocytes. Fresh human erythrocytes were obtained by centrifuging the whole blood at 3000 revolutions per minute (rpm) for 10 min. Hemolysis was monitored by measuring the absorbance of hemoglobin released due to cell lysis at 414 nm.

Hemolytic activity is conventionally used to measure the cytotoxicity or biocompatibility of antimicrobial polymers [31,32]. Figure 3 shows that the abilities of Polymer C_4_, C_6_, C_8_ to induce hemolysis were positively related to the alkyl chain length. The Polymer C_8(benzene)_ had relative lower hemolysis ability than the Polymer C_8_. Although the well-known Polymer C_6_ (Polyhexamethylene guanidine hydrochloride, PHGH) has been suggested to be used as a harmless disinfectant to fight against methicillin-resistant *Staphylococcus aureus*, which also is one of six high-priority dangerous ESKAPE pathogens, it is noted that PHGH induced 10% hemolysis rate. Polymer C_8_ with the highest bactericidal activity has very high hemolysis rate, which changed in the range of 0–90%.

### 3.3. Dye Leakage of Model POPC Lipid Vesicles Mimicking the Eukaryotic Membrane

**Figure 4:**
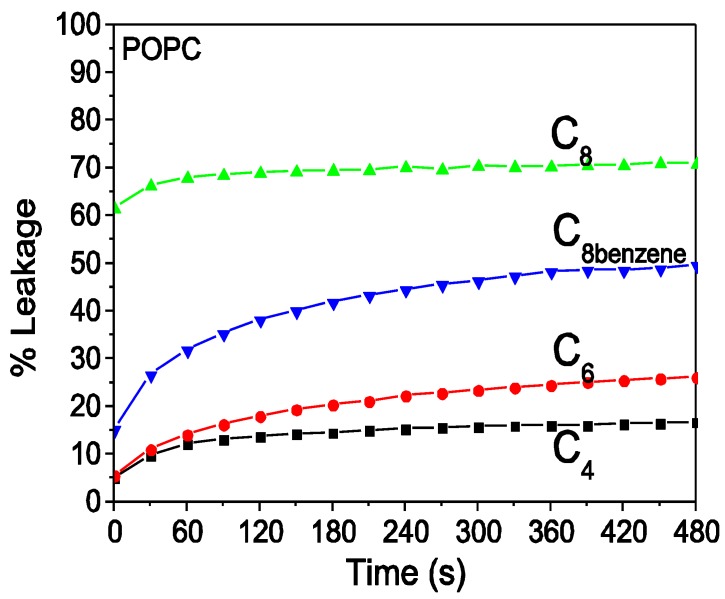
Calcein fluorescence dye leakage of 1-palmitoyl-2-oleoyl-sn-glycero-3-phosphocholine (POPC) vesicles after exposure to Polymer C_4_, C_6_, C_8_, and C_8(benzene)_. Calcein-loaded LUVs of 20 μM final concentration. Each oligoguanidine polymer with a final concentration of 10 μg/mL. An excitation wavelength of 490 nm and an emission wavelength of 515 nm with 5-nm bandwidth slits.

The leakage, determined by the fluorescence measurement, of calcein dye encapsulated in the model membrane prepared by the POPC lipid has been previously applied to monitor the membrane interaction of the cationic antimicrobial polymer [22,26,33]. POPC vesicles have net zero surface charge because POPC lipids have one negatively charged head group and one positively charged head group. Figure 4 shows that four oligoguanidine polymer analogs added to the anionic POPC vesicles induce calcein dye leakage, and each leakage trace had an extremely slow increase trend throughout the 480 s time range. Polymer C_4_ and C_6_ induced 10–20% leakage rate, but Polymer C_8_ and C_8(benzene)_ lead to 45–70% leakage rate. The leakage rate induced by Polymer C_8(benzene)_ was relatively lower compared to that induced by Polymer C_8_. Generally, the ability of Polymer C_4_, C_6_, and C_8_ to induce dye leakage was positively correlated with the carbon chain length of the alkyl group of the polymer’s repeat unit.

### 3.4. Thermodynamics of Polymer-Model Membrane Interactions

To further characterize the differences of polymer-lipid membrane interaction caused by the different structures of alkyl chain which responded to different thermodynamics, isothermal titration calorimetry (ITC) experiments were performed.

**Figure 5:**
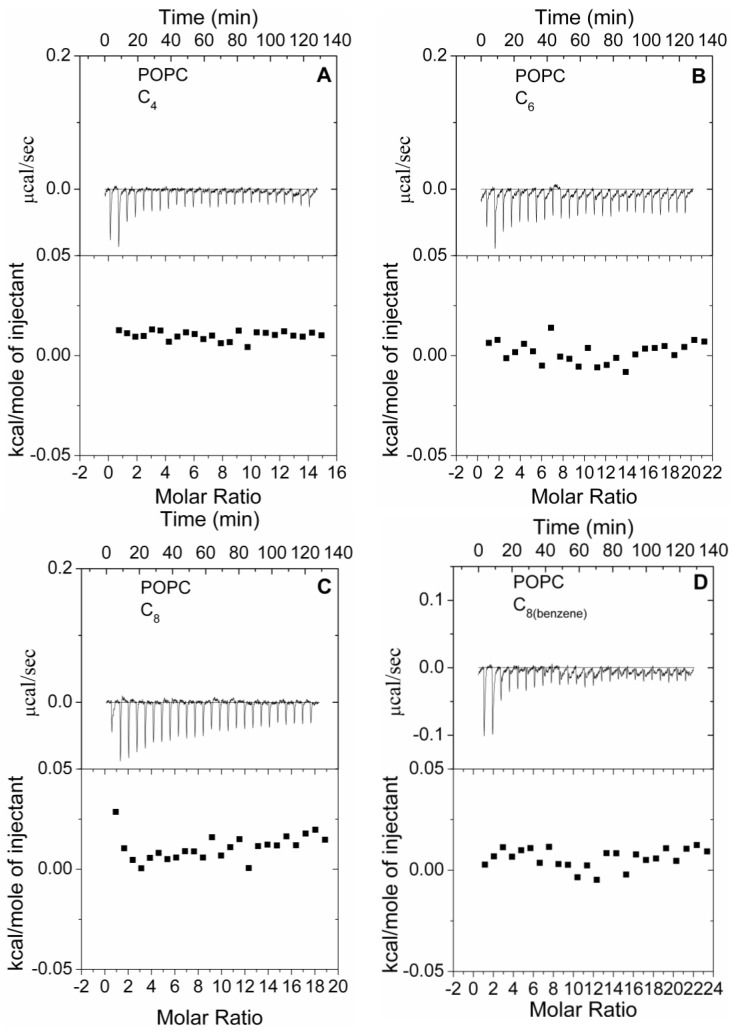
Titration calorimetry of POPC vesicles into Polymer C_4_, C_6_, C_8_, C_8(benzene)_ solutions at 30 °C. (**A**) Polymer C_4_; (**B**) Polymer C_6_; (**C**) Polymer C_8_; (**D**) Polymer C_8(benzene)_. Each oligoguanidine polymer solution at a concentration of 75 μg/mL. Large unilamellar vesicle (LUV) suspension (15 mM) was used. The lower curve in each case represents the heat of reaction (measured by peak integration) as a function of the lipid/polymer molar ratio.

The ITC traces obtained by titrating zwitterionic POPC LUVs into four polymers solutions at 30 °C were showed in Figure 5. For each of Polymer C_4_ (Figure 5A), C_6_ (Figure 5B), C_8_ (Figure 5C), the absolute value of the exothermic effect still showed a positive relationship with the length of the alkyl chain of the polymer. Comparison with the Polymer C_8(benzene)_ (Figure 5D)_,_ the Polymer C_8_ induced more exothermic effect. The thermodynamics of the polymer-POPC membrane agreed well with their hemolytic properties. But it is noteworthy that, for every one of four polymers, each injection produced very weak exothermic effect which slowly decreased with consecutive injections. The poorly unsaturated titration curves indicated that the four polymers had much lower affinity for zwitterionic POPC vesicles. The too low affinity and too little energy change for four polymers made it impossible to derive the binding constant for the equipment. Therefore, only qualitative conclusions could be made in these cases.

### 3.5. Toxicological Studies

When the administered dose was 200 and 500 mg/kg, all animals survived and no abnormalities in life were revealed throughout 14 days. Three (two female and one male) mice died on the account of the treatment with 800 mg/kg. The necropsy revealed no obvious pathological abnormalities with the exception of dead animals. These mice had light lungs, flat liver and spleen, and light mucous membranes. These results showed that both POGH and PHGH had an oral median lethal dose (LD_50_) ranging between 500 mg of polymer/kg of body weight and 800 mg/kg, as shown in the Table 1. Based on the chemical hazard classification of the Environmental Protection Agency (Washington, DC, USA), both POGH and PHGH had Category III toxicity with a LD50 range of 500 to 5000 mg/kg (oral administration to rats), slightly toxic.

## 4. Discussion

It is clear that there is a lack of related papers about antimicrobial polymers targeting bacterial membrane that are mainly focused on their synthesis methods, antimicrobial activities and mechanisms, and their biocompatibility evaluation on the phospholipid membrane of eukaryotic cell [34].

In the present research, four oligoguanidine polymer analogs exhibited gradually increased biocidal activity with the increasing carbon chain length of the alkyl group. Correspondingly, four guanidine polymer analogs exhibited gradually increased hemolytic activity and dye leakage rate with the increasing alkyl chain length, which were demonstrated by their ITC traces. As shown in Figure 3 and Figure 4, respectively, the induced hemolytic activity and dye leakage by Polymer C_6_, C_8_, C_8(benzene)_ implied these polymers have the possibility to damage the phospholipid membrane of the eukaryotic cell to different degrees. We paid attention to Polymer C_6_ (Polyhexamethylene guanidine hydrochloride, PHGH) and Polymer C_8_ (Polyoctamethylene guanidine hydrochloride, POGH) because of their high bactericidal activities against MRSA [19,21] and MDR-PA [19] of the high-priority dangerous ESKAPE pathogens in clinical practice.

Systematic studies have claimed that the length of the alkyl chains, the positive charge, and the spatial relationship between the alkyl chains and the cationic groups modulate antimicrobial and hemolytic activity of synthetic cationic-amphiphilic polymers [35]. The guanidinium groups of the polymer play important roles in its antimicrobial activity because of the ability of guanidinium to bind the phosphate group of the bacterial phospholipid membrane. The ability of the guanidinium unit to bind the phosphate group could be ascribed to guanidinium’s planar and rigid structure, and to its geometrical complementarity to this oxoanion, which allows the formation of a two-point hydrogen bonding chelate motif [36,37]. This hydrophile-lipophile balance was achieved by varying the side chain hydrophobicity or cationic charge in a variety of cationic-amphiphilic polymers [38]. Generally, the polymers with optimal amphiphilicity, which were obtained by changing the length of the alkyl chains, the positive charge, and their spatial relationship, selectively target the bacterial membranes instead of mammalian membranes [38,39].

The linear alkyl group of the guanidine polymer has flexible extensibility and is favorable to partition into the hydrophobic core region of the phospholipid membrane. It is probable that the hydrophobicity change of the linear alkyl group of the polymer plays relatively more important roles in the polymer-membrane interactions compared to the positively charge of the polymer. So for Polymer C_4_, C_6_, C_8_, the longer alkyl chain meant stronger hydrophobicity, which led to the polymer’s increasing ability to partition into the hydrophobic core region of the phospholipid membrane of both the bacteria and eukaryotic cell, although the eukaryotic membrane had zero charge. Subsequently, the antimicrobial activity, hemolytic activity and dye leakage of four polymers were determined. Because the appropriate alkyl chain length and charge, the Polymer C_6_ had the best hydrophile-lipophile balance, which led to its high antimicrobial activity and relatively low hemolytic activity. The rigid benzene ring of Polymer C_8(benzene)_ limited its partition into the lipid membrane compared to the flexible alkyl chain of Polymer C_8_. Therefore, Polymer C_8(benzene)_ had weak binding to membrane, which led to weaker antimicrobial activity than Polymer C_8_.

The observed exothermic effect and unsaturated titration curve of Polymer C_4_, C_6_, C_8_, C_8(benzene)_ by titrating zwitterionic POPC vesicles were drastically different from the endothermic effect and saturated titration curve of four polymers binding to anionic POPE/POPG vesicles, which mimic the bacterial membrane [22]. The comparison implied that Polymer C_4_, C_6_, C_8_, C_8(benzene)_ had much stronger binding ability to the bacterial membrane compared to the phospholipid membrane of the eukaryotic cell, which were probably determined by the charge difference. The heat effect detected by the ITC was the overall heat changes, and the electronic interactions were exothermic. When Polymer C_4_, C_6_, C_8_, C_8(benzene)_ were titrated in to the zwitterionic POPC vesicles, two hydrophobic regions from the polymer and the POPC membrane come in close contact, which liberated solvent molecules and led to a whole exothermic effect. However, these biocidal guanidine polymers, with a positive charge and enough conformational flexibility, exhibited strong binding affinity to the anionic POPE/POPG vesicles simulating the bacterial membrane, which suggested the energy released by the electronic interactions was not enough for the disruption of the permeability barrier of the lipid membrane, and additional energy absorbed from the system was necessary; subsequently, an overall endothermic effect was observed.

Cumulative reports suggested that Polymer C_6_ (PHGH) was probably a novel disinfectant tool to fight meticillin-resistant *Staphylococcus aureus*, which is one of six high-priority dangerous antibiotic-resistant ESKAPE pathogens [19,20,21]. In addition, the obvious advantage of Polymer C_6_ is that it could been covalently bound to polyurethanes [40] or bacterial cellulose [41] to prepare wound dressing materials with non-leaching permanent sterile surface, and bond to starch to prepare antimicrobial paper [42,43]. Therefore, the characteristic of Polymer C_6_, which could induce a slight hemolysis rate with 10% as the maximum value, should be considered in its industry application. The new synthesized Polymer C_8_ (POGH) showed a strong possibility to damage the phospholipid membrane of the eukaryotic cell.

## 5. Conclusions

In conclusion, the present study revealed that the Polymer C_4_, C_6_ (polyhexamethylene guanidine hydrochloride, PHGH), C_8_ (polyoctanethylene guanidine hydrochloride, POGH), and C_8(benzene)_ displayed gradually increased antimicrobial activity with the increased alkyl chain length. Correspondingly, four oligoguanidine polymer analogs exhibited gradually increased hemolytic activity and dye leakage with the increasing alkyl chain length. The thermodynamic curve of the polymer-POPC membrane indicated four polymers had weak affinity for zwitterionic POPC vesicles. Though Polymer C_6_ (PHGH) and Polymer C_8_ (POGH) showed good antimicrobial activities against antibiotic resistant bacteria, they induced hemolysis to different extents and both are in toxicity Category III with an LD_50_ range of 500 to 5000 mg/kg (slightly toxic), which should be considered in their potential industry applications.

## Figures and Tables

**Table 1:** Acute toxic effects of PHGH and POGH.

Polymer	Median Lethal Dose (LD_50_)
PHGH (Polymer C_6_)	500–800 mg/kg
POGH (Polymer C_8_)	500–800 mg/kg

## Abbreviations

Polymer C_4_	Polybutamethylene guanidine hydrochloride
Polymer C_6_ (PHGH)	Polyhexamethylene guanidine hydrochloride
Polymer C_8_ (POGH)	Polyoctamethylene guanidine hydrochloride
Polymer C_8(benzene)_	Poly(m-xylylene guanidine hydrochloride)
MDR-PA	Multidrug resistant *Pseudomonas aeruginosa*
MRSA	Methicillin-resistant *Staphylococcus aureus*
ITC	Isothermal titration calorimetry
